# Increased Prevalence of Symptoms of Rhinitis but Not of Asthma between 1990 and 2008 in Swedish Adults: Comparisons of the ECRHS and GA^2^LEN Surveys

**DOI:** 10.1371/journal.pone.0016082

**Published:** 2011-02-17

**Authors:** Anders Bjerg, Linda Ekerljung, Roelinde Middelveld, Sven-Erik Dahlén, Bertil Forsberg, Karl Franklin, Kjell Larsson, Jan Lötvall, Inga Sif Ólafsdóttir, Kjell Torén, Bo Lundbäck, Christer Janson

**Affiliations:** 1 The OLIN Studies, Department of Medicine, Sunderby Central Hospital of Norrbotten, Luleå, Sweden; 2 Department of Respiratory Medicine and Allergy, University of Umeå, Umeå, Sweden; 3 Department of Internal Medicine, Krefting Research Centre, University of Gothenburg, Göteborg, Sweden; 4 Centre for Allergy Research and Institute of Environmental Medicine, Karolinska Institutet, Stockholm, Sweden; 5 Environmental and Occupational Medicine, Department of Public Health and Clinical Medicine, Umeå University, Umeå, Sweden; 6 Department of Surgery, University of Umeå, Umeå, Sweden; 7 Lung and Allergy Research, Institute of Environmental Medicine, Karolinska Institutet, Stockholm, Sweden; 8 Occupational and Environmental Medicine, Sahlgrenska School of Public Health, University of Gothenburg, Göteborg, Sweden; 9 Department of Respiratory Medicine and Allergology, Sahlgrenska University Hospital, Göteborg, Sweden; 10 Department of Medical Sciences, Respiratory Medicine and Allergology, Uppsala University, Uppsala, Sweden; University College London - Institute of Child Health, United Kingdom

## Abstract

**Background:**

The increase in asthma prevalence until 1990 has been well described. Thereafter, time trends are poorly known, due to the low number of high quality studies. The preferred method for studying time trends in prevalence is repeated surveys of similar populations. This study aimed to compare the prevalence of asthma symptoms and their major determinants, rhinitis and smoking, in Swedish young adults in 1990 and 2008.

**Methods:**

In 1990 the European Community Respiratory Health Survey (ECRHS) studied respiratory symptoms, asthma, rhinitis and smoking in a population-based sample (86% participation) in Sweden. In 2008 the same symptom questions were included in the Global Allergy and Asthma European Network (GA^2^LEN) survey (60% participation). Smoking questions were however differently worded. The regions (Gothenburg, Uppsala, Umeå) and age interval (20–44 years) surveyed both in 1990 (n = 8,982) and 2008 (n = 9,156) were analysed.

**Results:**

The prevalence of any wheeze last 12 months decreased from 20% to 16% (p<0.001), and the prevalence of “asthma-related symptoms” was unchanged at 7%. However, either having asthma attacks or using asthma medications increased from 6% to 8% (p<0.001), and their major risk factor, rhinitis, increased from 22% to 31%. Past and present smoking decreased.

**Conclusion:**

From 1990 to 2008 the prevalence of obstructive airway symptoms common in asthma did not increase in Swedish young adults. This supports the few available international findings suggesting the previous upward trend in asthma has recently reached a plateau. The fact that wheeze did not increase despite the significant increment in rhinitis, may at least in part be due to the decrease in smoking.

## Introduction

Asthma is today a global public health issue, following a large increase in prevalence during the second half of the 20^th^ century.[1] The true magnitude of the increase however remains uncertain since many studies of time trends were either registry based, did not use general population samples, or compared prevalence numbers measured using different methodologies.[2], [3]

Accurate data on recent trends in disease are essential to both public health work and to the allocation of healthcare resources. Applying the same methodology to similar, representative samples from the population on two occasions is a reliable way to study time trends.[4] This method has become predominant in studies of children, several of which have demonstrated an unchanged prevalence of asthma in many European countries after year 2000.[5], [6] Somewhat surprisingly this repeated survey method has not been applied to adult populations to the same extent.

Repeated survey studies of Scandinavian adults demonstrated a prevalence increase until the 1990s.[7]–[9] A recent prevalence study suggested no further increase in asthma in West Sweden after 1990, when compared with older studies.[10] The only two recent repeated survey studies have reported a stable prevalence of adult asthma during the 1990s in Italy and the United Kingdom.[11], [12] Due to this scarcity of repeated surveys reaching into the 21^st^ century, further studies are needed.

The prevalence of respiratory symptoms, asthma, rhinitis and smoking was studied in 1990 in a large, population-based sample aged 20–44 years in three regions of Sweden as part of the European Community Respiratory Health Survey (ECRHS).[13] In 2008 the same questions were included in the Global Allergy and Asthma European Network survey (GA^2^LEN), which was mailed to 45.000 adults in Sweden. Using these two databases, the aim of the present study was to compare the prevalence of respiratory symptoms, asthma, rhinitis and smoking among Swedish young adults in 2008 with that in 1990.

## Materials and Methods

### Study areas

Three regions were surveyed: Gothenburg, Uppsala and Umeå. Gothenburg is located at the west coast and is Sweden's second largest city. It is the most densely populated study area with the highest pollution rates, and a mild and humid climate. Uppsala is a medium-sized university city with low industrial pollution and an inland climate. Umeå, the region capital of Northern Sweden, comprises both urban and sparsely populated rural areas. Industrial pollution is low and the climate is sub-arctic.

### The 1990 and 2008 surveys

The multinational ECRHS study of asthma symptoms, atopy, bronchial hyper-responsiveness and risk factors for asthma, was carried out in 1990.[14] The Swedish participation included a random sample of 10800 adults aged 20–44 years in Gothenburg, Uppsala and Umeå, identified using the Department of Statistics of the respective county council offices.[13] In Gothenburg the Hisingen area was surveyed. The ECRHS questionnaire included seven questions related to asthma (below) and five questions related to chronic bronchitis, and has been published in full.[13] It was translated and back-translated to minimise linguistic bias. Along with the questionnaire the study subjects received an invitation letter and a prepaid response envelope. Up to three reminders were subsequently mailed to non-responders. The final participation rate was 86%. The Ethics Committees of Gothenburg University, Uppsala University and Umeå University, and the Swedish Data Protection Board approved of the study.

The GA^2^LEN network of excellence was launched in 2004 and includes leading European research centres in the field of allergic diseases and asthma in 22 countries.[15] The GA^2^LEN survey, carried out in 2008, aimed to assess the prevalence of allergic disease (asthma, rhinitis, eczema) across Europe. Sweden participated with four centres: Gothenburg, Stockholm, Uppsala and Umeå. A random sample of 15,000 subjects from Gothenburg and 10,000 subjects from each of the other three cities aged 16–75 years were selected using civil registries. The questionnaire, in addition to the seven ECRHS questions on asthma and rhinitis also included questions related to chronic bronchitis, chronic obstructive pulmonary disease (COPD), obstructive sleep apnea syndrome (OSAS), environmental and workplace exposures, physical activity, and cardiovascular comorbidities. Up to three reminders were mailed. Sixty percent participated. The Regional Ethical Committee at Uppsala University approved of the Swedish participation in the GA^2^LEN survey, and of the present ECRHS-GA^2^LEN comparison study. In both surveys the information letter stated that the data would be stored and used for research purposes. Participants that answered the questionnaire were thus seen as having consented to participate in the study. This approach was approved of by the Ethics Committees both in 1990 and 2008.

### Questionnaires and definitions

The ECRHS questions are today widely used and have been validated against bronchial hyper responsiveness, spirometry and clinical evaluation by a panel of clinicians.[16], [17] The GA^2^LEN questionnaire included seven ECRHS core questions related to asthma and rhinitis, which have been published in full.[13] Only definitions of special relevance for the present paper are listed below.

Any wheeze: “Have you had wheezing or whistling in your chest at any time in the last 12 months?”

Wheeze with breathlessness: “Have you been at all breathless when the wheezing noise was present?”

Wheeze without a cold: “Have you had this wheezing or whistling when you did not have a cold?”

Asthma attacks: “Have you had an attack of asthma in the last 12 months?”

Asthma medications: “Are you currently taking any medicine (including inhalers, aerosols or tablets) for asthma?”

Rhinitis: “Do you have any nasal allergies including hay fever?”

“Asthma-related symptoms”: Any wheeze and wheeze with breathlessness and wheeze without a cold.

“Current asthma”: “Do you have asthma?” and either or both of asthma attacks and use of asthma medications.

Ever smoking, 1990: “Are you an ex-smoker? (quit smoking for more than one year).”

Current smoking, 1990: “Do you smoke? (answer yes even if you only smoke a few cigarettes or pipes per week or if you quit smoking for less than one year).”

Ever smoking >12 months, 2008: “Have you ever smoked one or more cigarettes per day for more than one year?”

Current smoking, 2008: “…If so, have you at all smoked during the last month?”

### Statistical analyses

The present study of time trends was limited to the centres (Gothenburg, Uppsala, Umeå) and age interval (20–44 years) surveyed both in 1990 and 2008. Questionnaires where information on any question was missing were eliminated, after which 8982 (4643 female) subjects in 1990 and 9156 (5202 female) subjects in 2008 remained for analysis.

For prevalence comparisons the two-sided chi^2^ test was used, with a p<0.05 significance level. Prevalence analyses were stratified for study centre and sex, respectively. For risk analyses multiple logistic regression was used to calculate Odds Ratios (OR) and 95% confidence intervals (CI). Interactions were tested by inclusion of interaction terms (e.g. study year*sex) in the model. All analyses were performed using SPSS Statistics ver. 11.5 (SPSS Inc, Chicago, USA). Adjusted population attributable fractions (aPAF) were calculated from the regression coefficients obtained by multiple logistic regression, following the method described by Eide et al. [18] and using STATA 9.1 (STATA Corp, Texas, USA).

## Results

### Trends in prevalence 1990 to 2008

**Figure 1:**
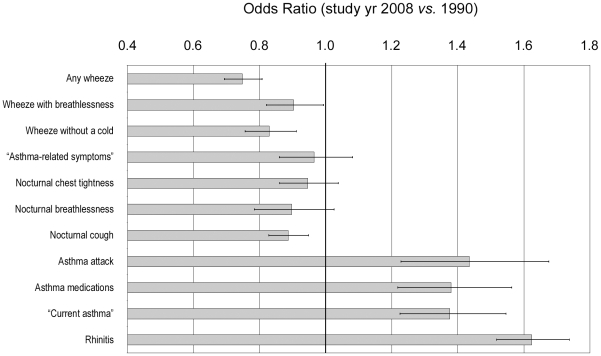
Adjusted odds ratios of study year 2008 *vs.* 1990. Multiple logistic regression analysis was performed with adjustment for age (20–27, 28–35, 36–44 years), sex and study centre. 95% confidence intervals are included.

The prevalence of any wheeze decreased in all centres from 20.3% (95% CI 19.4–21.1%) to 16.1% (15.4–16.9%), p<0.001 (Table 1). This was most pronounced in Gothenburg, where also wheeze with breathlessness and wheeze without a cold decreased statistically significantly. The prevalence of “asthma-related symptoms” remained at 7% and did not change statistically significantly, p = 0.40. Nocturnal symptoms declined significantly in Gothenburg and were unchanged in Uppsala and Umeå. However, the prevalence of “current asthma” increased from 6.0% (5.5–6.5%) to 8.0% (7.4–8.5%), p<0.001, since both asthma attacks and asthma medication use increased statistically significantly in all three centres. A multivariate model adjusting for age, sex and study centre confirmed these general trends in prevalence (Figure 1). In general, the prevalence trends were similar in men and women (Table 2).

**Table 1:** Prevalence (%) of respiratory symptoms in the last twelve months and of smoking, in 1990 and 2008, by study centre.

		All				Göteborg			Uppsala			Umeå		
		1990	2008	Mean diff. *	*P*	1990	2008	*P*	1990	2008	*P*	1990	2008	*P*
Wheeze	Any	20.3	16.1	−4.2	*<0.001*	22.5	15.7	*<0.001*	18.6	15.7	*0.005*	19.9	17.1	*0.005*
	with breathlessness	11.2	10.2	−1.0	*0.035*	11.9	9.4	*0.001*	10.3	10.4	*0.947*	11.3	11.1	*0.812*
	without a cold	12.1	10.2	−1.9	*<0.001*	13.1	9.4	*<0.001*	11.2	9.8	*0.102*	12.2	11.8	*0.620*
“Asthma-related symptoms”		7.2	6.9	−0.3	*0.396*	7.3	6.1	*0.051*	6.6	7.1	*0.522*	7.7	7.9	*0.802*
Woken by														
	chest tightness	11.2	10.9	−0.3	*0.474*	14.2	10.7	*<0.001*	9.4	10.6	*0.119*	10.4	11.3	*0.274*
	breathlessness	5.3	4.9	−0.4	*0.239*	6.9	5.0	*0.002*	4.8	4.5	*0.653*	4.4	5.1	*0.262*
	cough	26.6	24.7	−1.9	*0.003*	27.7	20.7	*<0.001*	25.3	27.1	*0.116*	26.9	27.8	*0.449*
Asthma attack		3.2	4.4	+1.3	*<0.001*	3.0	3.7	*0.130*	3.1	4.5	*0.007*	3.3	5.4	*<0.001*
Asthma medications		5.2	6.9	+1.7	*<0.001*	4.6	6.1	*0.009*	4.6	7.0	*<0.001*	6.2	7.9	*0.011*
“Current asthma”		6.0	8.0	+1.9	*<0.001*	5.5	6.9	*0.002*	5.7	8.1	*<0.001*	6.8	9.3	*<0.001*
Rhinitis		21.6	30.9	+9.4	*<0.001*	21.7	31.5	*<0.001*	22.0	30.9	*<0.001*	21.1	30.2	*<0.001*
Smoking														
	ever	50.1	-			55.2	-		48.3	-		47.4	-	
	current	35.3	-			42.2	-		33.6	-		31.1	-	
	>12 months ever	-	26.0			-	30.7		-	25.2		-	20.1	
	last month	-	11.7			-	14.5		-	11.1		-	8.3	

*Mean difference 2008 *vs.* 1990, absolute percentage.

**Table 2:** Prevalence of respiratory symptoms in the last twelve months and of smoking, in 1990 and 2008, by sex.

		Women				Men			
		1990n = 4643	2008n = 5202	Mean diff.*	*P*	1990n = 4339	2008n = 3954	Mean diff.*	*P*
Wheeze	Any	21.0	16.6	−4.4	*<0.001*	19.5	15.5	−4.0	*<0.001*
	with breathlessness	11.9	10.8	−1.1	*0.084*	10.4	9.4	−1.0	*0.144*
	without a cold	11.8	10.4	−1.4	*0.032*	12.5	10.0	−2.5	*<0.001*
“Asthma-related symptoms”		7.2	7.1	−0.1	*0.906*	7.3	6.6	−0.7	*0.239*
Woken by									
	chest tightness	11.0	12.0	+1.0	*0.133*	11.4	9.4	−2.0	*0.003*
	Breathlessness	5.6	5.2	−0.4	*0.348*	5.0	4.6	−0.4	*0.394*
	Cough	32.4	30.4	−2.0	*0.029*	20.4	17.1	−3.2	*<0.001*
Asthma attack		3.4	5.2	+1.8	*<0.001*	3.0	3.5	+0.5	*0.204*
Asthma medications		5.4	7.2	+1.8	*<0.001*	4.9	6.4	+1.5	*0.003*
“Current asthma”		6.3	8.5	+2.2	*<0.001*	5.7	7.2	+1.5	*0.005*
Rhinitis		21.2	30.5	+9.3	*<0.001*	21.9	31.5	+9.5	*<0.001*
Smoking									
	Ever	51.2	-			48.8	-		
	current	36.7	-			33.8	-		
	>12 months ever	-	27.8			-	23.6		
	Last month	-	12.2			-	10.6		

In 1990, ever smoking and current smoking was reported by 50.1% (49.0–51.1%) and 35.3% (34.3–36.3%) respectively, while in 2008 ever smoking for more than 12 months and any smoking last month were reported by 26.0% (25.1–26.9%) and 11.7% (11.0–12.4%) respectively (Table 1). Smoking prevalence was the highest in Gothenburg. Among never-smokers the prevalence of any wheeze and “asthma-related symptoms” did not change significantly from 1990 to 2008 (Table 3). The prevalence of “current asthma” among never-smokers increased from 6.3% (5.6–7.0%) to 7.8% (7.2–8.4%), p<0.01, and this increase was most pronounced in females and in Uppsala.

**Table 3:** Prevalence (%) of any wheeze, “asthma-related symptoms” and “current asthma” in never-smokers, in 1990 and 2008 respectively.

		Any wheeze			“Asthma-related symptoms”			“Current asthma”		
		1990	2008	*P*	1990	2008	*P*	1990	2008	*P*
All		14.4	14.2	*0.704*	5.7	6.2	*0.284*	6.3	7.8	*0.003*
Sex	Female	14.4	14.5	*0.922*	5.9	6.4	*0.511*	6.0	7.9	*0.007*
	Male	14.5	14.0	*0.595*	5.5	6.0	*0.361*	6.6	7.7	*0.106*
Age	20–24 yrs	15.0	14.3	*0.546*	6.9	6.8	*0.924*	7.9	8.4	*0.616*
	25–29 yrs	13.7	14.2	*0.694*	5.1	6.1	*0.292*	6.1	7.7	*0.119*
	30–34 yrs	13.2	13.0	*0.876*	5.6	6.3	*0.504*	4.7	7.1	*0.023*
	35–39 yrs	15.0	15.2	*0.907*	5.2	6.3	*0.350*	5.4	8.1	*0.027*
	40–44 yrs	15.4	14.8	*0.753*	4.5	5.0	*0.678*	5.9	7.4	*0.258*
Centre	Gothenburg	15.0	12.8	*0.070*	4.9	4.7	*0.807*	5.8	6.6	*0.317*
	Umeå	15.5	15.7	*0.862*	6.4	7.4	*0.201*	7.6	9.2	*0.075*
	Uppsala	12.9	14.5	*0.171*	5.6	6.8	*0.138*	5.3	7.9	*0.002*

### Asthma and wheeze with rhinitis

**Figure 2:**
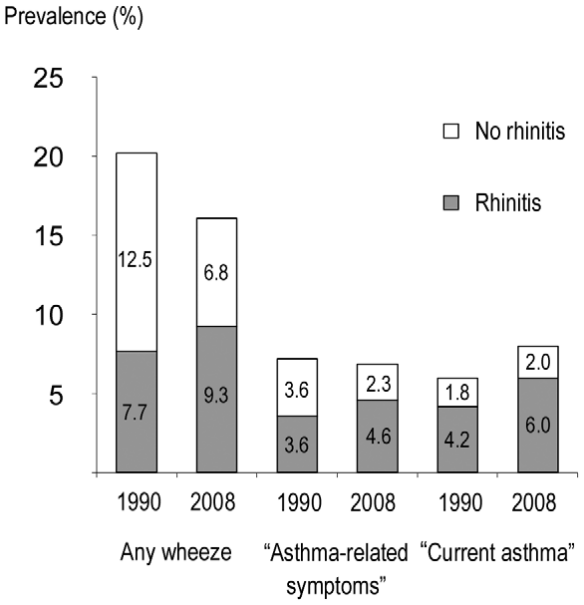
Prevalence stratified by rhinitis. Prevalence (%) of any wheeze, “asthma-related symptoms” and “current asthma” with and without rhinitis, respectively, in 1990 and 2008.

The prevalence of rhinitis rose significantly in all centres, from 21.6% (20.7–22.4%) to 30.9% (30.0–31.9%), p<0.001 (Table 1). The prevalence of any wheeze, “asthma-related symptoms” and “current asthma” with concurrent rhinitis increased, each p<0.01 (Figure 2). Meanwhile, the prevalence of any wheeze and “asthma-related symptoms” without concurrent rhinitis decreased, each p<0.01.

**Figure 3:**
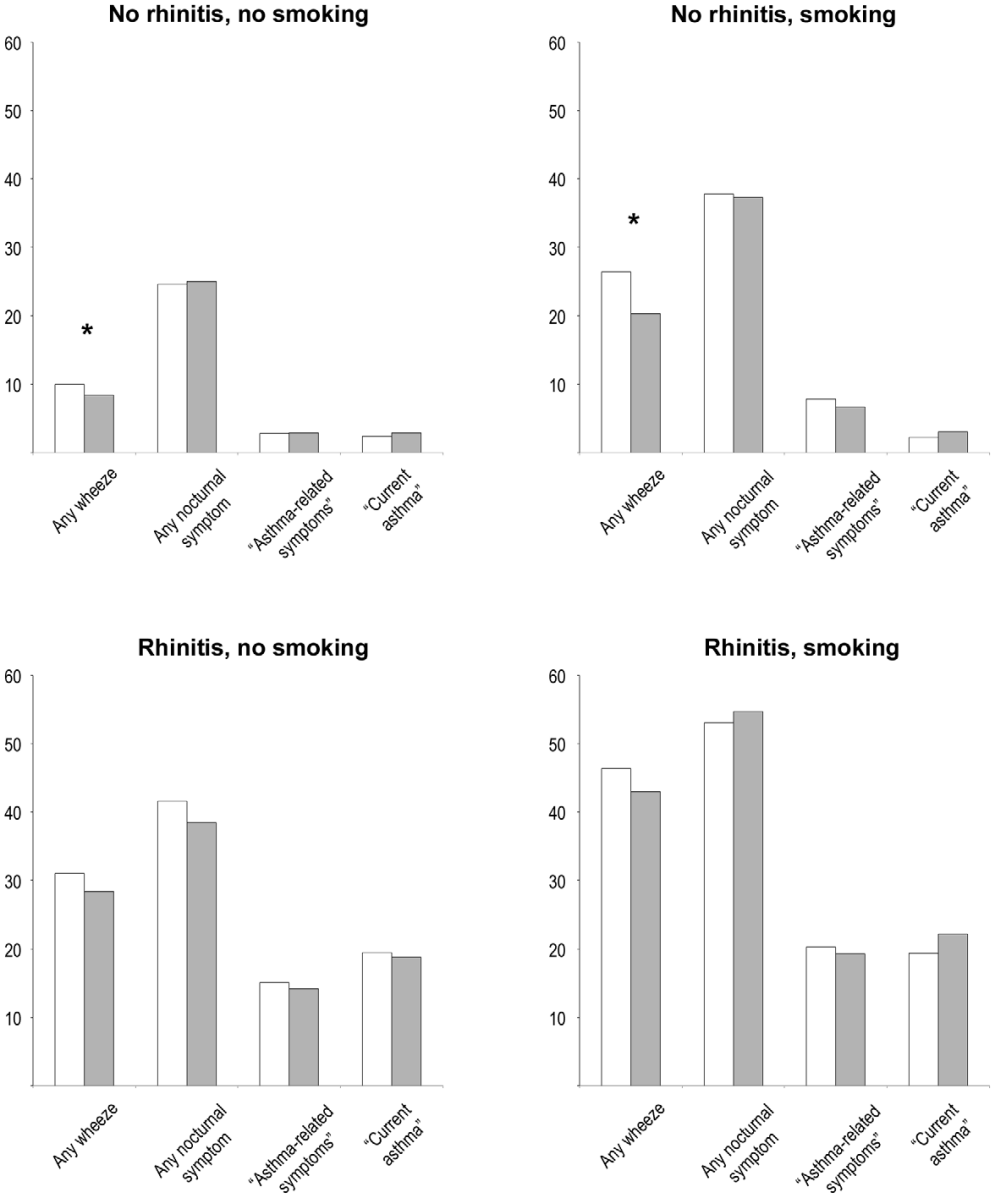
Prevalence in four mutually exclusive risk groups. Prevalence (%) of any wheeze, “asthma-related symptoms”, “current asthma” and of any nocturnal symptom, in four subgroups based on current smoking (yes/no) and rhinitis (yes/no). White bars: Study year 1990. Grey bars: Study year 2008. * denotes *p<0.05*.

Four mutually exclusive risk groups were studied: non-smokers without rhinitis, non-smokers with rhinitis, smokers without rhinitis and smokers with rhinitis (Figure 3). From 1990 to 2008 there were only small changes in the prevalence of any wheeze, any nocturnal symptom, “asthma-related symptoms” and “current asthma” within each group. The prevalence of nocturnal symptoms was around 25% in non-smokers without rhinitis, and above 50% in smokers with rhinitis. Any wheeze was reported by 30% of subjects with rhinitis, and by 40% of smokers with rhinitis.

### Risk factor analysis

Multivariate analysis demonstrated that the major risk factors for any wheeze were rhinitis, OR 3.65 (95% CI 3.36–3.96) and current smoking, OR 2.71 (2.47–2.98) (Table 4). This was similar to “asthma-related symptoms”, where rhinitis had OR 4.71 (4.18–5.30) and current smoking OR 2.08 (1.81–2.39). “Current asthma” was strongly associated with rhinitis, OR 9.02 (7.92–10.3) but showed no association with smoking, and was significantly less common in subjects aged over 30 years. Living in Umeå was associated with the highest risk for all three outcomes (ORs 1.2).

The majority of associations were of similar strength 1990 and 2008 (Table 5). However, there was a downward trend over time in the association of living in Gothenburg with wheeze and “asthma-related symptoms” (p = 0.02, p = 0.09 for the interaction term study year*Gothenburg). Risk associations by smoking status are presented in tables 6 and 7. Notably, in 2008 female sex was a significant risk factor among ever smokers only (Table 7).

**Table 4:** Risk factors, including study year, for “asthma-related symptoms”, “current asthma” and any wheeze by multiple logistic regression analysis.

		“Asthma-related symptoms”	“Current asthma”	Any wheeze
		OR (95% CI)	OR (95% CI)	OR (95% CI)
Study year 2008 (vs. 1990)		0.98 (0.86–1.11)	1.12 (0.98–1.27)	**0.83 (0.76–0.90)**
Female sex		1.03 (0.92–1.16)	**1.19 (1.06–1.34)**	**1.09 (1.00–1.17)**
Age (vs. 20–24 yrs)				
	25–29 yrs	0.92 (0.77–1.10)	0.86 (0.72–1.03)	1.01 (0.90–1.15)
	30–34 yrs	0.90 (0.75–1.09)	**0.74 (0.61–0.89)**	0.93 (0.82–1.06)
	35–39 yrs	0.92 (0.76–1.11)	**0.79 (0.65–0.95)**	1.02 (0.90–1.17)
	40–44 yrs	0.95 (0.79–1.15)	**0.78 (0.64–0.94)**	1.07 (0.94–1.22)
Centre (vs. Uppsala)				
	Umeå	**1.20 (1.04–1.38)**	**1.24 (1.07–1.44)**	**1.15 (1.04–1.27)**
	Gothenburg	0.91 (0.79–1.06)	0.87 (0.75–1.02)	1.06 (0.96–1.17)
Rhinitis		**4.71 (4.18–5.30)**	**9.02 (7.92–10.3)**	**3.65 (3.36–3.96)**
Smoking (vs. never)				
	Ever	1.10 (0.91–1.31)	1.07 (0.90–1.28)	**1.16 (1.02–1.31)**
	Current	**2.08 (1.81–2.39)**	1.09 (0.93–1.28)	**2.71 (2.47–2.98)**

All variables in the table were included in the model. Associations are presented as Odds Ratios (OR) with 95% confidence intervals (95% CI). Statistically significant associations in bold text.

**Table 5:** Risk factors for “asthma-related symptoms”, “current asthma” and any wheeze by multiple logistic regression analysis, in 1990 and 2008 respectively.

		“Asthma-related symptoms”		“Current asthma”		Any wheeze	
		1990 OR (95% CI)	2008 OR (95% CI)	1990 OR (95% CI)	2008 OR (95% CI)	1990 OR (95% CI)	2008 OR (95% CI)
Female sex		0.98 (0.83–1.15)	1.08 (0.91–1.28)	1.15 (0.96–1.39)	**1.22 (1.04–1.44)**	1.08 (0.97–1.21)	1.09 (0.96–1.22)
Age (vs. 20–24 yrs)							
	25–29 yrs	0.91 (0.71–1.16)	0.95 (0.74–1.21)	0.80 (0.61–1.04)	0.91 (0.73–1.15)	1.00 (0.85–1.18)	0.98 (0.82–1.16)
	30–34 yrs	0.85 (0.66–1.09)	0.90 (0.70–1.16)	**0.65 (0.49–0.87)**	0.83 (0.65–1.06)	0.99 (0.84–1.16)	0.90 (0.75–1.08)
	35–39 yrs	0.85 (0.66–1.09)	1.05 (0.81–1.35)	0.77 (0.58–1.01)	0.93 (0.73–1.19)	0.98 (0.83–1.16)	1.07 (0.89–1.28)
	40–44 yrs	1.11 (0.86–1.43)	0.82 (0.62–1.09)	0.75 (0.56–1.01)	0.77 (0.59–1.01)	1.08 (0.91–1.29)	1.06 (0.87–1.28)
Centre (vs. Uppsala)							
	Umeå	**1.23 (1.01–1.50)**	1.17 (0.95–1.44)	**1.29 (1.04–1.60)**	1.21 (0.99–1.48)	**1.14 (1.00–1.30)**	1.16 (0.995–1.35)
	Gothenburg	1.03 (0.84–1.27)	**0.81 (0.66–0.998)**	0.96 (0.76–1.22)	**0.82 (0.67–0.996)**	**1.18 (1.03–1.35)**	0.95 (0.82–1.09)
Rhinitis		**4.44 (3.76–5.24)**	**5.05 (4.25–6.00)**	**10.2 (8.45–12.4)**	**8.06 (6.77–9.60)**	**3.30 (2.94–3.71)**	**4.06 (3.61–4.56)**
Smoking (vs. never)							
	Ever	0.98 (0.74–1.29)	1.21 (0.96–1.54)	1.18 (0.90–1.56)	1.00 (0.79–1.26)	1.17 (0.98–1.40)	1.16 (0.98–1.40)
	Current	**2.14 (1.79–2.55)**	**1.92 (1.53–2.40)**	1.05 (0.85–1.29)	1.21 (0.95–1.54)	**2.84 (2.52–3.20)**	**2.43 (2.08–2.85)**

All variables in the table were included in the model. Associations are presented as Odds Ratios (OR) with 95% confidence intervals (95% CI). Statistically significant associations in bold text.

**Table 6:** Risk factors in never-smokers and ever smokers in 1990 by multiple logistic regression analysis.

		“Asthma-related symptoms”		“Current asthma”		Any wheeze	
		Never-smokers	Ever smokers	Never-smokers	Ever smokers	Never-smokers	Ever smokers
		OR (95% CI)	OR (95% CI)	OR (95% CI)	OR (95% CI)	OR (95% CI)	OR (95% CI)
Female sex		0.94 (0.72–1.21)	1.04 (0.74–1.48)	1.13 (0.88–1.46)	1.16 (0.89–1.51)	1.01 (0.85–1.20)	1.13 (0.99–1.30)
Age (vs. 20–24 yrs)							
	25–29 yrs	0.74 (0.51–1.06)	1.04 (0.74–1.48)	0.76 (0.54–1.07)	0.89 (0.58–1.38)	0.91 (0.71–1.16)	1.01 (0.81–1.26)
	30–34 yrs	0.78 (0.54–1.14)	0.85 (0.60–1.21)	**0.55 (0.38–0.82)**	0.82 (0.54–1.25)	0.85 (0.66–1.10)	0.95 (0.76–1.18)
	35–39 yrs	0.78 (0.52–1.16)	0.85 (0.61–1.19)	0.68 (0.45–1.01)	0.92 (0.61–1.37)	1.03 (0.79–1.34)	0.85 (0.68–1.05)
	40–44 yrs	0.71 (0.45–1.12)	1.22 (0.88–1.70)	0.81 (0.53–1.23)	0.79 (0.51–1.22)	1.12 (0.85–1.48)	0.90 (0.73–1.13)
Centre (vs. Uppsala)							
	Gothenburg	0.92 (0.65–1.29)	1.18 (0.91–1.54)	1.15 (0.82–1.62)	0.81 (0.58–1.12)	1.23 (0.99–1.54)	**1.21 (1.03–1.43)**
	Umeå	1.14 (0.84–1.53)	1.24 (0.95–1.62)	**1.46 (1.09–1.97)**	1.11 (0.81–1.53)	**1.24 (1.01–1.52)**	1.04 (0.88–1.24)
Rhinitis		**6.00 (4.60–7.79)**	**3.44 (2.77–4.28)**	**9.71 (7.42–12.7)**	**10.9 (8.28–14.4)**	**4.01 (3.37–4.77)**	**2.65 (2.27–3.10)**

All variables in the table were included in the model. Associations are presented as Odds Ratios (OR) with 95% confidence intervals (95% CI). Statistically significant associations in bold text.

**Table 7:** Risk factors in never-smokers and ever smokers in 2008 by multiple logistic regression analysis.

		“Asthma-related symptoms”		“Current asthma”		Any wheeze	
		Never-smokers	Ever smokers	Never-smokers	Ever smokers	Never-smokers	Ever smokers
		OR (95% CI)	OR (95% CI)	OR (95% CI)	OR (95% CI)	OR (95% CI)	OR (95% CI)
Female sex		0.96 (0.78–1.18)	**1.40 (1.03–1.91)**	1.00 (0.83–1.21)	**2.34 (1.64–3.33)**	0.99 (0.86–1.15)	**1.30 (1.06–1.61)**
Age (vs. 20–24 yrs)							
	25–29 yrs	0.88 (0.66–1.17)	1.13 (0.70–1.82)	0.87 (0.67–1.12)	1.14 (0.70–1.84)	0.97 (0.79–1.19)	0.92 (0.66–1.28)
	30–34 yrs	0.90 (0.67–1.21)	0.87 (0.53–1.43)	0.79 (0.60–1.05)	1.00 (0.61–1.63)	0.86 (0.70–1.07)	0.83 (0.59–1.16)
	35–39 yrs	0.86 (0.63–1.18)	1.39 (0.87–2.22)	0.88 (0.66–1.17)	1.13 (0.69–1.84)	1.02 (0.82–1.27)	0.96 (0.69–1.34)
	40–44 yrs	**0.67 (0.47–0.97)**	1.07 (0.66–1.74)	0.80 (0.59–1.10)	0.74 (0.43–1.24)	1.00 (0.79–1.27)	0.99 (0.71–1.38)
Centre (vs. Uppsala)							
	Gothenburg	**0.68 (0.53–0.88)**	1.12 (0.79–1.60)	0.83 (0.66–1.05)	0.77 (0.53–1.11)	0.87 (0.73–1.04)	1.15 (0.90–1.46)
	Umeå	1.14 (0.89–1.46)	1.19 (0.79–1.79)	1.23 (0.98–1.55)	1.15 (0.76–1.74)	1.13 (0.95–1.35)	1.20 (0.90–1.60)
Rhinitis		**5.87 (4.73–7.27)**	**3.76 (2.80–5.03)**	**7.70 (6.29–9.42)**	**9.24 (6.51–13.1)**	**4.43 (3.84–5.10)**	**3.23 (2.64–3.95)**

All variables in the table were included in the model. Associations are presented as Odds Ratios (OR) with 95% confidence intervals (95% CI). Statistically significant associations in bold text.

**Figure 4:**
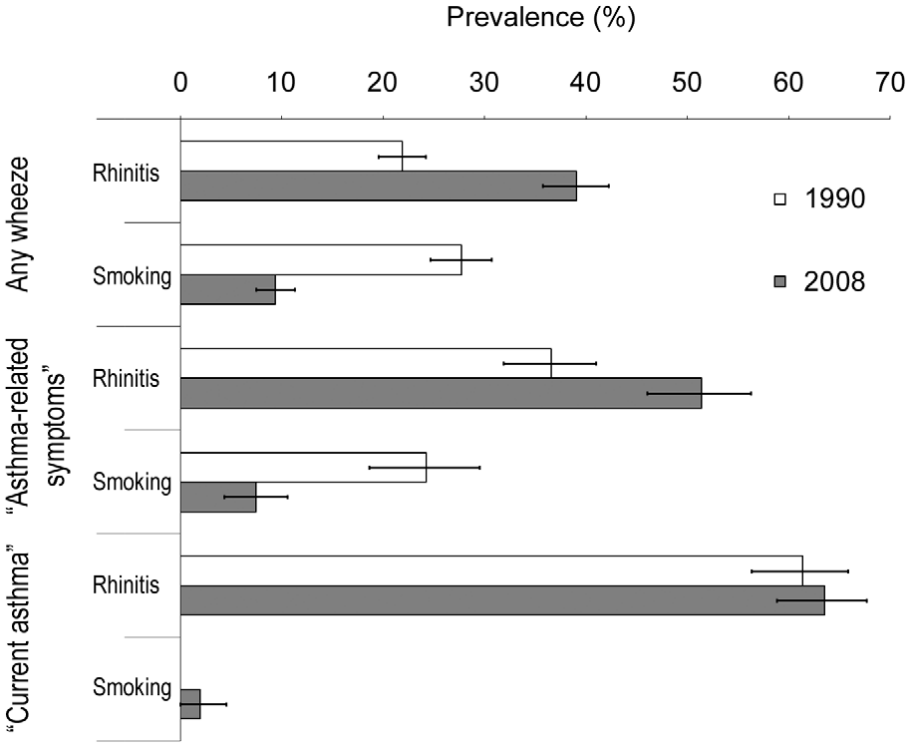
Adjusted population attributable fractions (aPAF). Fractions (%) including their 95% confidence intervals of any wheeze, “asthma-related symptoms” and “current asthma” in the population attributable to rhinitis and smoking, adjusted also for age (20–27, 28–35, 36–44 years), sex and study centre by multiple logistic regression analysis.

Adjusted population attributable fractions (aPAF) were calculated from multivariate ORs and risk factor prevalence (Figure 4). For any wheeze, in 1990 rhinitis and current smoking each had aPAF around 25% whereas in 2008, aPAF was 40% for rhinitis and 10% for smoking. This pattern was seen also for “asthma-related symptoms”, only with greater aPAF of rhinitis in both surveys. For “current asthma”, the aPAF of rhinitis was around 60% in both surveys while that of smoking was negligible.

## Discussion

This large, population-based repeated survey study found no further increase after 1990 in wheezing indices and “asthma-related symptoms” among Swedish young adults. In fact, the prevalence of any wheeze decreased from 1990 to 2008, whereas the prevalence of asthma attacks and the use of asthma medication increased slightly. The prevalence of rhinitis, on the other hand, increased considerably. Smoking, although surveyed differently in 1990 and 2008, seemed to decrease.

### Strengths and limitations

The present study benefitted from applying the same standardised ECRHS core questions to two comparable large general population samples surveyed 18 years apart. Asking about symptoms is less likely biased by health care practice and diagnostic activity than is e.g. asthma diagnoses. Two findings indicated that the core questions targeted similar respiratory symptoms both years. First, the associations of respiratory symptoms with smoking and rhinitis were very similar in 1990 and 2008. Second, the prevalence of other respiratory symptoms among subjects with any wheeze, “asthma-related symptoms” and “current asthma” were also comparable in both surveys (data not shown).

The age spans and regions surveyed were similar in 1990 and 2008. The choice of age span minimises the risk of misclassification with COPD, however, the findings cannot be readily generalised to older adults. The three study areas were chosen so as to represent the geographical (north *vs.* south, inland *vs.* coastal, west *vs.* east coast) and demographical (large city, medium-sized city and a smaller community) features of Sweden.[13] In 1990, the Gothenburg survey was carried out in the area of Hisingen, which had lower mean incomes and a higher proportion of immigrants than the rest of the city.[13] In 2008 a general Gothenburg population sample was invited instead. However, the major trends in any wheeze, “asthma-related symptoms” and rhinitis were seen also in Umeå and Uppsala, and in another study of Gothenburg.[10] Moreover, adjustment for differences in the study populations did not change these observations (Figure 1).

A decreased participation, as seen in 2008 compared to 1990, may have lead to overestimation of symptom prevalence [19] in the latter survey. The resulting false-positive prevalence trend would however not contradict our main finding that prevalence did not increase. Also, a recent Swedish study, which included a subsample from the present study, found no evidence of such participation bias of respiratory symptoms.[20]

### Trends in symptoms of asthma

Asthma is a heterogeneous disease. We thus sought to mirror the panorama of respiratory morbidity from any wheeze to more specific “asthma-related symptoms”.[11], [13] The outcome “asthma-related symptoms” reflects both non-allergic and allergic asthma and bronchial hyper-reactivity [13] and was closely related to smoking and rhinitis. Any wheeze, in contrast, seemingly had other significant determinants, as suggested by the low aPAFs of smoking and rhinitis. The negative time trend in any wheeze, OR 0.83 (0.76–0.90), when adjusted for rhinitis and ever smoking probably reflected changes in exposures not surveyed, e.g. air pollution or workplace exposures. The decrease in any wheeze, although seen in all study centres, was most pronounced in Gothenburg. Analogously, the other two wheezing indices were unchanged in Umeå and Uppsala, and decreased in Gothenburg. The differences between 1990 and 2008 in the Gothenburg population composition, as discussed above, may have led to an overestimation of the negative trend. On the contrary, lower participation in 2008 may have biased the prevalence slightly upwards.[19] Taking these uncertainties into consideration, our conservative conclusion is that there was no evidence of a continuation of the previous upward trend in obstructive symptoms.

In contrast to any wheeze and the other wheezing indices, the prevalence of “current asthma” increased slightly. There are several possible explanations for this. First, one would expect a real increase in the prevalence of “current asthma” driven by the large increase in rhinitis, to which current asthma was strongly associated. Second, by its definition “current asthma” is affected by secular trends in prescription patterns and by the labelling of respiratory symptoms as “asthma”. In Sweden awareness of asthma has increased among physicians and in the community [6], [10] and this may have contributed to the observed increase. This inherent limitation and the fact that other obstructive symptoms did not increase or even decreased, argue that a true increase in asthma prevalence cannot be directly inferred from the increase in “current asthma”. If anything, it may reflect a slight change in the spectrum of respiratory symptoms between 1990 and 2008.

In the Scandinavian countries, including Sweden, a rise in asthma was seen between the 1970s and 1990s.[7]–[9], [21] Recently, a subset comparison indicated no increase in asthma symptoms in West Sweden,[10] and the present study confirms these findings outside that region as well. In Swedish schoolchildren, no increase in asthma symptoms was seen 1996–2006.[6] These indications of a break of the previous upward trend in Sweden warrants further up-to-date studies in the other Scandinavian countries.

In the UK, asthma in adults has been studied more thoroughly.[22], [23] A review concluded that the prevalence plateaued during the 1990s after increasing since the 1970s,[12] a conclusion, however, in part based on registry studies and studies of selected populations.[22] The present study corroborates these findings despite the higher asthma prevalence in the UK. Hypothetically, this could indicate that trends in prevalence correlate better to events in time than to absolute prevalence, analogous to recent observations in children.[5], [6], [12] Hopefully, further studies will determine whether in fact a plateau in prevalence has developed simultaneously in children and adults.

Registry-based studies have indicated a level asthma prevalence during the 1990s also in Mexico, Canada and the US.[24]–[26] In an Italian repeated survey study the prevalence of asthma attacks, wheeze and “asthma-related symptoms” was unchanged during the 1990s, breaking a previous upward trend.[11], [27] Regrettably, the Italian study did not include trends in risk factors. Unlike the Italian authors we do not believe that increased medication explains the decrease in any wheeze, since also the prevalence of either having wheeze or using medications decreased in our study, 21.2%–17.8%, p<0.001.

Taken together, our results in conjunction with previous findings provide increasing evidence that asthma in adults has reached a plateau in prevalence in several Westernised countries after 1990. This relatively simultaneous development in geographically very different locations is in itself an important observation, as it would argue against some aetiological factors, e.g. certain allergens, as a major explanation. However, until more studies include trends in determinants of asthma, researchers can only speculate about the underlying aetiologies.

### Trends in rhinitis and smoking

The mean annual increase in rhinitis was 0.52% in our study, similar to 0.41% in Italy 1991–1998.[11] An increase in rhinitis was also seen in a Danish study which included serological tests for allergic sensitisation.[28] In contrast, in Melbourne, Australia, the prevalence of rhinitis was unchanged 1990–1999.[29] However, this could be reflect a saturation effect, since the prevalence in Melbourne in 1990 was almost twice that in our study. In 1990 the sensitisation rate was 76% among adults with rhinitis in the Swedish ECRHS centres. Provided this proportion did not change 1990–2008 our results may suggest an increase in sensitisation among Swedish adults. Without objective testing this, however, remains a speculation.

Although lower participation and the more stringent wording in 2008 of the questions about smoking may have biased the trend in smoking prevalence downward, our results are very similar to several recent Swedish population-based studies [6], [10] and also to Swedish official census data.[30] This is firm evidence of a real decrease in smoking, which may have been related to recent smoking restrictions, raised tobacco taxes and information campaigns.

For a multifactorial disease with complex cause-consequence relationships such as asthma, exact attributable fractions cannot be calculated from single risk factor odds ratios. Risk factor associations may also be biased: Physicians may be more prone to diagnose rhinitis in asthmatic subjects, or to label wheeze among smokers as COPD. We however used aPAF, which takes both risk factor prevalence and strength of association into account, as a crude proxy to detect large-scale trends in risk factors in the population. Using the approximations of risk in our study it seems that, at a population level, the importance of smoking decreased while that of rhinitis increased. These changes were mainly due to changed prevalence of smoking and rhinitis, which is less subject to association bias, above. We speculate that the decrease in smoking is one reason why the prevalence of wheeze and “asthma symptoms” did not increase to the same extent that did rhinitis.

### Conclusion

In conclusion, the present study demonstrated that symptoms of airway obstruction have not increased in prevalence since 1990 in Swedish young adults. The observed decrease in smoking may be one explanatory factor. A moderate increase was however seen for asthma attacks and use of asthma medications. Despite the possible bias from changed awareness, this finding may indicate a slight shift in respiratory symptom expression, related to the significant increase in rhinitis. Although the studies are still few, there seems to be a general trend toward a plateau in asthma in Westernised countries. The fact that this seemingly has occurred simultaneously in children and adults, and in several parts of the world, may provide an important clue in understanding the development of the asthma epidemic.
